# SARS-CoV-2 Omicron: a new challenge for pandemic and vaccine

**DOI:** 10.1038/s41392-022-01088-7

**Published:** 2022-07-05

**Authors:** Xiantao Zhang, Hui Zhang, Xin He

**Affiliations:** Institute of Human Virology, Department of Pathogen Biology and Biosecurity, and Key Laboratory of Tropical Disease Control of Ministry of Education, Zhongshan School of Medicine, Sun Yat-sen University, 510080 Guangzhou, China

**Keywords:** Translational research, Infection

In a recent article published in *Nature*, Shuai et al. demonstrated a significantly reduced replication capacity and diminished pathogenicity of SARS-CoV-2 Omicron, the latest variant of concern (VOC), mainly due to the impaired Spike protein (S protein) cleavage, reduced efficiency in utilizing co-factor TMPRSS2.^1^

**Fig. 1:**
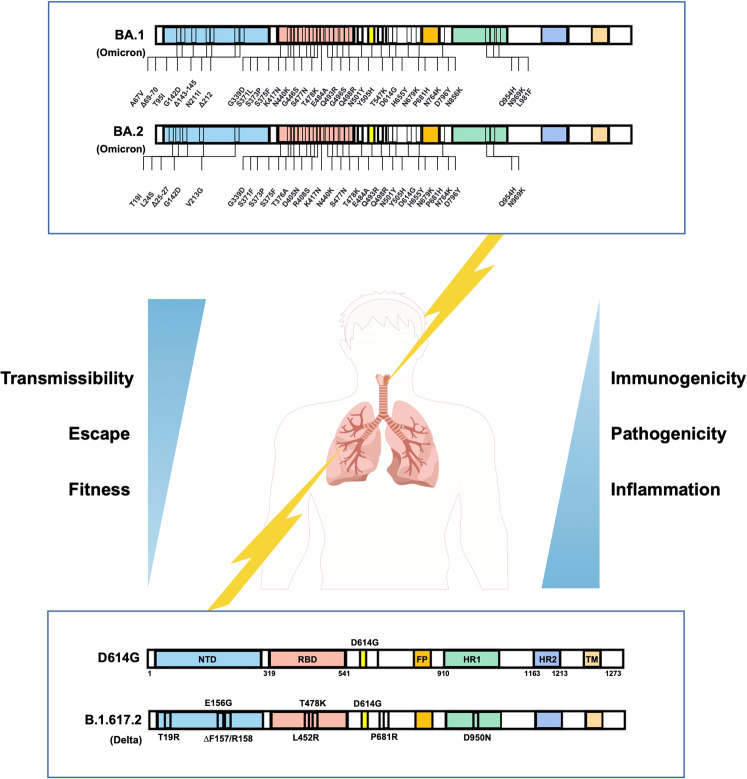
Omicron and its subvariants evolved to be adaptation and balance between transmission, pathogenicity and immune escape. Compared to early VOCs, such as D614G or Delta strains (bottom part), Omicron and its subvariants have more than thirty mutations in the Spike (top part). The Omicron-infected and replicated more in the upper respiratory tract, but the early VOCs attacked the lung seriously. The new variant Omicrons have an enhanced infectivity and immune evasion capabilities, with low inflammation and mild symptoms

The replication of Omicron was attenuated in both Calu3 and Caco2 cells.^1^ The mechanistic studies showed that Omicron utilized the TMPRSS2 receptor less efficiently compared to other VOC strains. Omicron replication was also significantly attenuated in both upper and lower airways of infected K18-hACE2 mice compared to Delta or early HKU-001a (including D614) strains. Moreover, Omicron infection resulted in minimal weight loss and death. To sum up, they demonstrated a significantly reduced viral replication and pathogenesis of Omicron, accompanied with a much lower inflammation effect (Fig. 1).

Meanwhile, it has been found that Omicron Spike has a higher affinity for ACE2 than Delta.^2^ Replications of Omicron and Delta in human nasal mucosal epithelial cells are similar. However, Omicron replicated much less in lower respiratory tract organs, alveolar cells and intestinal cells than Delta. Omicron inefficiently utilizes TMPRSS2 for membrane fusion upon entry into cells, suggesting more dependence on Cathepsins for Omicron. The impaired S1/S2 cleavage and the inability to utilize TMPRSS2 illustrate the reduced virulence of Omicron. The mice infected with Omicron had an almost 100% survival rate compared to Delta and other VOCs.^3^ The virulence in the upper respiratory tract (nose and bronchi) and lungs of hACE2 mice were very slight, which did not cause weight loss and fever. Consistently, Omicron infection caused less pro-inflammatory cytokine release, as well as low T-cell activation in the lung. A significantly reduced pneumonia and pathogenicity in Omicron-infected hamster compared to Delta or early VOCs.^4^

An interesting finding is the significantly reduced utilization of TMPRSS2 of the Omicron, whose entry was insensitive to Camostat, a TMPRSS2 inhibitor. The change from TMPRSS2-depedent to TMPRSS2-indepedent entry of Omicron, suggests a new evolutional advantage that further enhances the infectivity. In addition, the TMPRSS2-indepedence of Omicron also indicates possible immune escape from the unnoticed neutralizing antibodies against the interaction between TMPRESS2 and Spike. Another interesting point is that the Omicron may hijack some other cofactors to accomplish cell entry. Therefore, it is interesting to identify the potential host co-factor for targeted therapy.

Omicron has intense immune escape from the sera after early VOCs infection, due to the heavy mutations in Spike.^2^ However, a third dose of mRNA vaccination remains partly efficient to neutralize Omicron. The sera of unvaccinated patients or mice infected with Omicron could only block Omicron virus, without cross-variant neutralizing activity to early VOCs, possibly caused by the lower and narrower cross-reacted antibody response elicited by virus infection.^3^ The titer of the neutralizing antibody in the sera from Omicron-infected unvaccinated patient was at least five-fold lower than early VOCs for their specific protection. Particularly, the cross-variant neutralizing activities between BA.1 and BA.2 infected-sera were also low for each other.

So far, the Omicron continuously evolved to generate more than twenty new subvariants with similar characteristics, including BA.2, BA.4, BA.5, and so on.

Omicron BA.2 has been predominant in at least 68 countries. The transmissibility coefficient of Omicron BA.2 increased 1.4-fold compared to BA.1. BA.2 performed more capable of replicating in the nasal mucosa compared to BA.1, which resulted in a significantly efficient formation of syncytia.^5^ It is probable that Omicron subvariants with Delta-like mutations, such as BA.2.12.1, BA.4, and BA.5 with L452R/Q, would spread more rapidly and are therefore likely to emerge to be the predominant. Remarkably, as the evolution of Omicron subtypes aggress, Omicron seems to obtain the characteristics of influenza antigenic drift and increases the fitness, indicating a similar epidemiological trend of seasonal influenza but with severe mortality.

Omicron has more than thirty mutations in Spike protein compared to the early HKU-001a strain, which may reach an ultimate version at the current stage, and may take a long time for other VOC stains to emerge to substitute. From the molecular epidemiological data of the last 5 months, Omicron also emerges to undergo recovered mutations, such as the BA.2.12.1, BA4/5 based on Omicron, or a recombination of Omicron with early VOCs.

It is meaningful to pay more attention to the impact of Omicron plus some individual mutations, such as mutation at L452 or E484, which may further endow new VOCs with severe transmissibility, pathogenicity and immune escape. We also need to focus on the immune recognition and response of the human host to Omicron. One issue is that three doses of vaccinations designed from early strain elicited broader neutralization than Omicron-specific vaccine booster vaccination, possibly due to the weaker immunogenicity, in consistent with the lower immune response and inflammation during the Omicron infection.

In conclusion, Omicron and its subvariants have got extreme adaptation and balance between transmission, pathogenicity and immune escape. Omicron may still be prevalent for a period, with a significant challenge for the herd immunity and new vaccine development.
